# Total and High Molecular Weight Adiponectin Levels and Prediction of Cardiovascular Risk in Diabetic Patients

**DOI:** 10.1155/2015/545068

**Published:** 2015-05-05

**Authors:** Dagmar Horáková, Kateřina Azeem, Radka Benešová, Dalibor Pastucha, Vladimír Horák, Lenka Dumbrovská, Arnošt Martínek, Dalibor Novotný, Zdeněk Švagera, Milada Hobzová, Dana Galuszková, Vladimír Janout, Sandra Doněvská, Jana Vrbková, Helena Kollárová

**Affiliations:** ^1^Department of Preventive Medicine, Faculty of Medicine and Dentistry, Palacký University Olomouc, Hněvotínská 3, 775 15 Olomouc, Czech Republic; ^2^Clinic of Internal Medicine, University Hospital Ostrava, 17. Listopadu 1790, 708 52 Ostrava-Poruba, Czech Republic; ^3^Department of Sports and Exercise Medicine, Faculty of Medicine and Dentistry, Palacký University Olomouc, I.P. Pavlova 6, 775 15 Olomouc, Czech Republic; ^4^Department of Clinical Biochemistry and Immunogenetics, University Hospital Olomouc, I.P. Pavlova 6, 775 15 Olomouc, Czech Republic; ^5^Department of Clinical Biochemistry, University Hospital Ostrava, 17. Listopadu 1790, 708 52 Ostrava-Poruba, Czech Republic; ^6^Department of Respiratory Medicine, University Hospital Olomouc, I.P. Pavlova 6, 775 15 Olomouc, Czech Republic; ^7^Department of Transfusion Medicine, University Hospital Olomouc, I.P. Pavlova 6, 775 15 Olomouc, Czech Republic; ^8^Institute of Molecular and Translational Medicine, Faculty of Medicine and Dentistry, Palacký University Olomouc, Hněvotínská 3, 775 15 Olomouc, Czech Republic

## Abstract

The study aimed at assessing the potential use of lower total and HMW adiponectin levels for predicting cardiovascular risk in patients with type 2 diabetes mellitus (T2DM). Concentrations of total adiponectin or high molecular weight (HMW) adiponectin decrease in association with the development of metabolic dysfunction such as obesity, insulin resistance, or T2DM. Increased adiponectin levels are associated with a lower risk for coronary heart disease. A total of 551 individuals were assessed. The first group comprised metabolically healthy participants (143 females, and 126 males) and the second group were T2DM patients (164 females, and 118 males). Both total adiponectin and HMW adiponectin in diabetic patients were significantly lower when compared with the group of metabolically healthy individuals. There was a weak monotonic correlation between HMW adiponectin levels and triglycerides levels. Binary logistic regression analysis, gender adjusted, showed a higher cardiovascular risk in diabetic persons when both total adiponectin (OR = 1.700) and HMW adiponectin (OR = 2.785) levels were decreased. A decrease in total adiponectin levels as well as a decrease in its HMW adiponectin is associated with a higher cardiovascular risk in individuals with T2DM. This association suggests that adiponectin levels may be potentially used as an epidemiological marker for cardiovascular risk in diabetic patients.

## 1. Introduction

Adiponectin, a protein produced by adipocytes, plays an important role in the metabolism of glucose and lipids. Adiponectin circulates in human serum as high molecular weight (HMW form) and low molecular weight (LMW form) oligomers. The ratio of the HMW form to the total adiponectin level is referred to as the adiponectin sensitivity index [1]. Experimental in vitro animal studies have shown that adiponectin affects many pathways potentially leading to coronary heart disease (CHD). Adiponectin also protects the vascular endothelium and has anti-inflammatory effects [2]. Results of numerous studies have confirmed the beneficial role of adiponectin in insulin sensitivity and cardiovascular (CV) disease prevention [3]. Increased adiponectin levels are associated with a reduced risk for CHD [4]. Decreasing adiponectin levels could potentially be used as a marker for CV risk in diabetic persons.

## 2. Methods

The study was a part of a Czech Ministry of Health Internal Grant Agency Project carried out at the Faculty of Medicine and Dentistry, Palacký University Olomouc, Czech Republic. Data were collected from January 2011 to April 2013. The study was approved by the Institutional Ethics Committee and informed consent was obtained from all participants. Two groups of subjects were enrolled. Group A (controls) comprised healthy individuals with no clinical or biochemical markers of metabolic disease. Group B (cases) consisted of patients with type 2 diabetes mellitus (T2DM) treated with oral antidiabetic drugs, most frequently metformin. None of TDM2 patients used thiazolidine. Healthy controls were recruited at the Department of Exercise Medicine and Cardiovascular Rehabilitation and Blood Transfusion Department of the University Hospital Olomouc. Diabetic patients were recruited at a diabetes outpatient center of the Department of Internal Medicine, University Hospital Ostrava.

CV risk criteria were as follows: a history of nonfatal myocardial infarction or clinically manifest atherosclerosis (coronary artery disease, cerebrovascular ischemic disease, and peripheral arterial disease) and/or hypolipidemic therapy in anamnesis. CV events in diabetic persons were confirmed in the medical records.

Laboratory analyses were carried out at the Department of Clinical Biochemistry, University Hospital Olomouc.

In all participants, waist circumference, body height and weight for body mass index (BMI) calculations, and blood pressure were measured. After 12-hour fasting, venous blood was collected.

### 2.1. Laboratory Analysis

Venous blood samples were drawn in the morning after a 12-hour fasting. After centrifugation, the serum was used for other analyses. For assessment of prothrombotic markers, venous blood was collected in 3.8% sodium citrate tubes and plasma was obtained after centrifugation.

Routine serum biochemical parameters were analyzed on Modular SWA (Roche, Basel, Switzerland) on the day of blood collection. Concentrations of adipokines and other special analytes were measured in sample aliquots stored at −80 (−20) °C for no longer than 6 months (see the following). Total cholesterol, triglycerides, and HDL cholesterol were determined enzymatically on the above analyzer. Concentrations of apolipoprotein B (ApoB) and ApoA1 were determined immunoturbidimetrically using the Tina-quant ApoB and ApoA1 kits (Roche). Glucose was determined using GOD-PAP method (Roche). All tests were performed using fresh sera on the day of blood collection.

Insulin was determined by the IRMA method using a commercially available kit (Immunotech, Marseille, France) and specific antibodies. Total adiponectin (one separate aliquot stored at −80°C until the day of analysis) was determined with the Human Adiponectin ELISA immunochemical kits (BioVendor Laboratory Medicine, Brno, Czech Republic) according to the manufacturer's instructions and after verification of methods. HMW adiponectin was determined from the same aliquot by ELISA using the HMW Adiponectin ELISA Kit (Otsuka Pharmaceutical, Tokyo, Japan).

### 2.2. Statistical Analysis

Statistical analysis was performed using the computing environment R (R Foundation for Statistical Computing, Austria; http://www.r-project.org/). Data were presented as basic robust (median) and nonrobust (mean, standard deviation) summary characteristics (Table 1). Monotonic dependence of total adiponectin or HMW adiponectin on triglycerides, ApoA1, or ApoB was quantified using Spearman's rank correlation coefficient (*r*); at the same time, its significant difference from zero was tested (Table 2). Gender adjusted univariate binary logistic regression model with continuous predictor variable (adiponectin or HMW-CV disease risk factors) was used to model the risk of CV disease (Table 3). The level of statistical significance was set at 5%.

The adiponectin sensitivity index was calculated as HMW adiponectin/total adiponectin ratio. The Homeostasis Model Assessment (HOMA IR) index was calculated as HOMA IR = fasting insulin (*μ*IU/mL) × fasting glucose (mmol/L)/22.5.

## 3. Results

Age, BMI, waist circumference, blood pressure, and metabolic parameters in both groups are shown in Table 1. Group A (healthy individuals) consisted of 269 participants (143 females and 126 males) with a mean age of 57.5 years (56.8 years in females and 55.8 years in males). In this group, the metabolic parameters showed normal values. The mean total adiponectin level was high at 10.6 mg/L (10.34 mg/L in females and 8.04 mg/L in males). The mean HMW adiponectin level was 5.2 mg/L (4.71 mg/L in females and 4.46 mg/L in males). The mean total adiponectin and mean HMW adiponectin levels were higher in females compared to males. The adiponectin sensitivity index was 0.45 in females and 0.55 in males. The Homeostasis Model Assessment (HOMA IR) index was low at 2.54 in females and 3.0 in males, suggesting normal insulin sensitivity. Group B (diabetic patients) comprised 282 individuals (164 females and 118 males) with a mean age of 59.8 years (62.1 years in females and 63.9 years in males). These participants were treated with oral antidiabetic drugs for T2DM. In all the studied parameters, Group B was different from healthy subjects. Group B members had obesity (BMI 32.03 in females and BMI 31.84 in males), hypertension (142/84 mmHg in females and 149/86 mmHg in males), hypercholesterolemia (5.14 mmol/L in females and 5.34 mmol/L in males), hypertriglyceridemia (1.98 mmol/L in females and 2.24 mmol/L in males), and hyperglycemia (8.39 mmol/L in females and 8.43 mmol/L in males) at the time of measurement. Their total adiponectin level was significantly lower (5.32 mg/L in females and 5.12 mg/L in males) as compared with the healthy females and males. Similarly, the mean HMW adiponectin level was significantly lower (2.92 mg/L in females and 3.03 mg/L in males) than that in healthy females and males. The adiponectin sensitivity index was 0.54 in females and 0.59 in males in Group B. Their HOMA IR index was 5.28 in females and 5.65 in males, a value typical for patients with insulin resistance. Table 2 shows weak monotonic dependence of total adiponectin or HMW adiponectin on triglycerides, ApoA1, or ApoB in females and males. Univariate binary logistic regression analysis, gender adjusted, was performed to predict the risk of CV disease. Subjects belonging to the risk group were identified according to the entry criteria for CV risk. Table 3 with results of the logistic regression analysis shows CV risk for both total adiponectin (OR 1.700) and HMW adiponectin (OR 2.785) levels. The results suggest that lower levels of total adiponectin or its HMW oligomer may predict the risk of CV disease in patients with T2DM.

## 4. Discussion

The discovery of adiponectin seems to be a landmark in the study of obesity and the related cardiometabolic diseases such as T2DM, atherosclerosis, and hypertension. Adiponectin circulates in serum at relatively high concentrations in metabolically healthy individuals. Today, its levels may be detected using available methods. Unlike some other adipose tissue proteins, adiponectin is of epidemiological interest due to the fact that its lower concentrations are associated with the development of some metabolic dysfunctions such as insulin resistance accompanied by accumulation of adipose tissue and development of obesity [5]. Adiponectin circulates in human plasma as oligomers of HMW (e.g., dodecamers and octadecamers) and LMW (e.g., trimers and hexamers). The HMW isoform of adiponectin seems to prevail, playing an important role in the metabolism of fatty acids and glucose and inflammation within intracellular posttranslational modifications [1]. Some recent epidemiological studies have shown that high HMW adiponectin levels may be associated with a CV risk reduction in middle-aged adults with high blood glucose levels [1]. For all these reasons, the question is whether lower HMW adiponectin levels may be used as a predictor for metabolic dysfunction. Also in the present group of metabolically healthy individuals, the total adiponectin level was high (F 10.34 mg/L and M 8.04 mg/L), almost double that in diabetic patients (F 5.32 mg/L and M 5.12 mg/L). Similarly, HMW adiponectin levels are substantially higher in healthy persons than in diabetics. The HMW adiponectin isoform has the largest share of the total adiponectin level. Biosynthesis of adiponectin multimers is a complex process involving extensive posttranslational modifications, with secretion from adipocytes being strictly controlled by the endoplasmic reticulum and modified according to the current metabolic condition of the cell. Cross-sectional studies have shown that levels of adiponectin forms are slightly affected by both age and gender. The main parameters influencing total adiponectin levels are accumulation of adipose tissue and a rise in BMI, glucose level, and renal function [6]. Elderly individuals and those with kidney disease were not included in the present study. Adipose tissue accumulation leading to the development of obesity that is accompanied by a prevailing proinflammatory state clearly leads to a decrease in both total adiponectin and HMW adiponectin levels [7]. Similarly, in diabetic patients participating in the present study, diabetes mellitus was accompanied by obesity and CRP levels were slightly raised as compared with metabolically healthy individuals. Experimental studies have agreed that adiponectin is a protein closely involved in the metabolism of glucose and lipids and inflammation [8, 9].

Adiponectin accelerates reverse cholesterol transport by increasing HDL cholesterol level through increased expression and secretion of ApoA1 [3]. Apolipoprotein A1 is the main protein of HDL particles and its levels are decreased in atherosclerosis. Diabetics in the present study had lower ApoA1 levels than healthy individuals. Moreover, diabetic patients have hypercholesterolemia and hypertriglyceridemia, potentially increasing their risk for the development of atherosclerosis. Previous studies support the hypothesis that a great deal of adiponectin effects on vascular endothelium may be mediated by metabolism of HDL cholesterol [10, 11]. High levels of both total adiponectin and HMW adiponectin are associated with a lower risk of CHD. This relationship is largely mediated by lipid metabolism, especially HDL cholesterol, as well as glucose and inflammation [3]. The association between individual lipoprotein particles and adiponectin levels may vary depending on the development of cardiovascular lesions; a decrease in adiponectin levels means a compensatory reaction. HMW adiponectin levels positively correlate with total cholesterol and HDL cholesterol levels [7]. The present study found a weak correlation between HMW adiponectin and ApoA1, particularly among healthy individuals. This supports the hypothesis that high HMW levels may reduce the risk for CV diseases in middle-aged adults, with a multivariate odds ratio of 0.33. However, the association was not observed in elderly individuals [12]. A meta-analysis of 12 prospective studies summarizing the effect of adiponectin on the CV risk showed that higher adiponectin levels were associated with a low risk of CHD (a pooled relative risk [RR] of 0.83). The protective effect was consistently shown in middle-aged males and females [4]. Significantly lower adiponectin levels together with increased CRP levels were demonstrated in a group of patients with myocardial infarction. Adiponectin has anti-inflammatory and antiatherogenic properties which may have a predictive value for the genesis and development of acute coronary syndrome [13]. A meta-analysis of 24 prospective studies assessing the pooled RR of adiponectin levels for CHD events yielded inconclusive results. The pooled RR of adiponectin for CHD events was not demonstrated (RR = 1.03) but subgroup analyses showed an increased risk for CHD recurrence and CV disease mortality [14]. Correlations between adiponectin levels and the HOMA scores have been consistently shown in adults. The results are practically identical irrespective of whether total adiponectin or HMW adiponectin is assessed [15, 16]. Adiponectin levels are lower in individuals with T2DM. This fact is partly explained by the presence of obesity and the phase of impaired glucose metabolism. The severity of dysglycemia in T2DM may be indirectly linked to adiponectin concentration. Adiponectin levels were even suggested as a potential clinical marker of therapeutic effectiveness [5]. In the present study, total adiponectin levels in diabetic patients were not significantly low. Even the median level did not drop below 4 mg/L which is a typical concentration for fully developed insulin resistance. This supports the hypothesis that treatment with oral antidiabetic drugs may increase adiponectin levels [1]. In the Diabetes Prevention Program, the therapeutic benefit of metformin treatment together with lifestyle changes was clearly associated with adiponectin levels [17, 18]. Cross-sectional studies on the relationship between glucose metabolism and adiponectin levels found that low adiponectin concentration may be associated with insulin resistance [19]. Insulin resistance and compensatory hyperinsulinemia might exert their atherogenic influence through decreased fibrinolytic activity. [20]. A decrease in adiponectin levels accompanied by obesity may be used to predict the development of diabetes mellitus [21]. Lower adiponectin levels increase the risk of the development of T2DM. The presence of T2DM increases the risk of the development of atherosclerosis and CV events. In diabetic persons, each increase in adiponectin levels by therapy or intervention was associated with a lower risk for CV events (OR 0.34) [22]. Key risk factors for CV diseases in adults diagnosed with diabetes mellitus are observed. In diabetic adults in the USA, for instance, classic risk factors such as blood pressure, glycated hemoglobin, and LDL cholesterol have significantly improved over a period of ten years [23]. The use of further potential biomarkers such as adiponectin levels for predicting the CV risk should be a matter of further research. Changes in both total adiponectin and HMW adiponectin levels may reflect metabolic dysregulation. With respect to this fact, the question is whether a decrease in total or HMW adiponectin levels may be used as a potential predictor of CV risk in T2DM patients. In the present study, binary logistic regression analysis showed a higher CV risk in T2DM individuals if both total and HMW adiponectin levels were decreased. There was not a major difference in odds ratios between total and HMW adiponectin levels. Potential use of adiponectin concentration measurements for predicting CV risk and its use as potential marker of response to therapy definitely need further verification and research.

## 5. Conclusion

A decrease in total adiponectin levels as well as a decrease in its high molecular weight oligomer (HMW adiponectin) is associated with a higher CV risk in individuals with T2DM. These findings suggest that lower concentrations of total adiponectin or its HMW oligomer may be used to predict CV risk in T2DM patients.

## Figures and Tables

**Table 1:** Basic metabolic and clinical characteristics of subjects.

Characteristics	Healthy		DM	
	Females	Males	Females	Males
	Mean ± SD (median)	Mean ± SD (median)	Mean ± SD (median)	Mean ± SD (median)
*N*	143	126	164	118
Age (years)	56.8 ± 10.2	55.8 ± 11.4	62.1 ± 9.2	63.9 ± 8.7
BMI (kg/m²)	25.3 ± 1.4 (25.5)	26.7 ± 3.5 (25.7)	32.03 ± 5.9 (30.83)	31.84 ± 5.2 (30.77)
Waist (cm)	78.5 ± 10.1 (79)	85.5 ± 12.0 (83)	112.5 ± 7.7 (105)	113.0 ± 11.8 (111)
BP systolic (mmHg)	122.53 ± 11.25 (120)	128.51 ± 13.79 (130)	142.17 ± 19.19 (140.5)	149.31 ± 17.07 (148)
BP diastolic (mmHg)	77.76 ± 8.90 (80)	79.1 ± 9.12 (80)	83.85 ± 11.40 (85)	85.92 ± 10.17 (86)
Total cholesterol (mmol/L)	4.97 ± 0.80 (4.95)	5.01 ± 1.05 (4.93)	5.14 ± 1.08 (5.08)	5.34 ± 1.02 (4.64)
Triglycerides (mmol/L)	1.3 ± 0.84 (1.08)	1.57 ± 1.04 (1.35)	1.98 ± 0.87 (1.81)	2.24 ± 1.21 (1.8)
HDL cholesterol (mmol/L)	1.71 ± 0.43 (1.69)	1.33 ± 0.33 (1.29)	1.32 ± 0.32 (1.27)	1.18 ± 0.31 (1.15)
LDL cholesterol (mmol/L)	2.68 ± 0.72 (2.62)	2.98 ± 0.83 (2.92)	3.17 ± 0.932 (3.16)	3.0 ± 0.799 (2.92)
Glucose (mmol/L)	5.16 ± 0.90 (4.9)	5.4 ± 1.52 (5.1)	8.39 ± 1.86 (8.22)	8.43 ± 1.76 (8.4)
Insulin (mIU/L)	10.64 ± 9.08 (7.5)	11.87 ± 12.73 (8.25)	13.97 ± 9.40 (10.7)	14.88 ± 10.62 (11.8)
ApoA-1 (g/L)	1.78 ± 0.31 (1.75)	1.6 ± 0.24 (1.48)	1.55 ± 0.25 (1.54)	1.45 ± 0.23 (1.42)
ApoB (g/L)	0.86 ± 0.20 (0.84)	0.96 ± 0.25 (0.94)	0.96 ± 0.23 (0.94)	0.92 ± 0.23 (0.9)
Total adiponectin (mg/L)	10.34 ± 4.59 (7.25)	8.04 ± 5.104 (6.68)	5.32 ± 1.47 (5.1)	5.12 ± 1.72 (4.87)
HMW adiponectin (mg/L)	4.71 ± 2.64 (4)	4.46 ± 2.83 (3.8)	2.92 ± 0.91 (2.9)	3.03 ± 1.109 (2.8)
HMW/total adiponectin ratio	0.45 ± 0.09 (0.56)	0.55 ± 0.06 (0.55)	0.54 ± 0.07 (0.57)	0.59 ± 0.07 (0.59)
HOMA IR	2.54 ± 2.50 (1.58)	3.0 ± 3.72 (1.99)	5.28 ± 3.90 (3.97)	5.65 ± 4.69 (4.25)

SD: standard deviation; M: male; F: female; BP: blood pressure; homeostasis model assessment.

HOMA IR = fasting insulin (IU/mL) × fasting glucose (mmol/L)/22.5.

**Table 2:** *P* values from the test of the significance of Spearman's rank correlation coefficient and the values of Spearman's rank correlation coefficient (*r*) between total adiponectin or HMW adiponectin and the variables.

	Females								Males							
	Healthy				DM				Healthy				DM			
	Total adiponectin		HMW adiponectin		Total adiponectin		HMW adiponectin		Total adiponectin		HMW adiponectin		Total adiponectin		HMW adiponectin	
	*r*	*P* value	*r*	*P* value	*r*	*P* value	*r*	*P* value	*r*	*P* value	*r*	*P* value	*r*	*P* value	*r*	*P* value
Triglycerides	0.187	0.081	0.205	0.056	−0.015	0.848	−0.065	0.394	0.059	0.445	0.062	0.419	−0.116	0.198	−0.075	0.409
ApoA-1	0.101	0.35	0.087	0.425	0.009	0.905	0.065	0.394	−0.016	0.84	−0.02	0.799	−0.027	0.762	−0.011	0.903
ApoB	0.106	0.33	0.125	0.249	0.093	0.222	0.002	0.978	−0.036	0.645	−0.03	0.697	−0.059	0.514	0.003	0.976

*P* < 0.05.

DM: diabetes mellitus.

**Table 3:** Univariate binary logistic regression of CVD risk factors, gender adjusted.

	Estimate	SE	*P* value	OR	95% CI
Total adiponectin	0.531	0.101	<0.001	1.700	1.411–2.096
HMW adiponectin	1.024	0.185	<0.001	2.785	1.980–4.088

Female = base category.

SE: standard error; OR: odds ratio; CI: confidence interval.
